# Wastewater Surveillance Data as a Complement to Emergency Department Visit Data for Tracking Incidence of Influenza A and Respiratory Syncytial Virus — Wisconsin, August 2022–March 2023

**DOI:** 10.15585/mmwr.mm7237a2

**Published:** 2023-09-15

**Authors:** Peter M. DeJonge, Carly Adams, Ian Pray, Melissa K. Schussman, Rebecca B. Fahney, Martin Shafer, Dagmara S. Antkiewicz, Adélaïde Roguet

**Affiliations:** ^1^Epidemic Intelligence Service, CDC; ^2^Wisconsin Department of Health Services; ^3^Division of Foodborne, Waterborne, and Environmental Disease, National Center for Emerging and Zoonotic Infectious Diseases, CDC; ^4^Career Epidemiology Field Officer Training Program, CDC; ^5^University of Wisconsin-Milwaukee, Milwaukee, Wisconsin; ^6^Wisconsin State Laboratory of Hygiene.

**Summary**

**What is already known about this topic?**

Wastewater surveillance is useful for tracking community SARS-CoV-2 levels, but its usefulness for tracking influenza and respiratory syncytial virus (RSV) is less understood.

**What is added by this report?**

During August 2022–March 2023, influenza and RSV were tracked using wastewater surveillance and emergency department (ED) visits in three Wisconsin cities. A positive correlation between the two surveillance systems was observed. Wastewater surveillance detected increases in influenza and RSV that preceded increases in ED visits by weeks and persisted beyond declines in associated ED visits for up to 3 months.

**What are the implications for public health practice?**

Incorporating wastewater surveillance into established surveillance systems might improve local preparedness and response to seasonal respiratory virus disease outbreaks.

## Abstract

Wastewater surveillance has been used to assist public health authorities in tracking local transmission of SARS-CoV-2. The usefulness of wastewater surveillance to track community spread of other respiratory pathogens, including influenza virus and respiratory syncytial virus (RSV), is less clear. During the 2022–23 respiratory diseases season, concentrations of influenza A virus and RSV in wastewater samples in three major Wisconsin cities were compared with emergency department (ED) visits associated with these pathogens. In all three cities, higher concentrations of influenza A virus and RSV in wastewater were associated with higher numbers of associated ED visits (Kendall’s tau range = 0.50–0.63 for influenza-associated illness and 0.30–0.49 for RSV-associated illness). Detections of both influenza A virus and RSV in wastewater often preceded a rise in associated ED visits for each pathogen, and virus material remained detectable in wastewater for up to 3 months after pathogen-specific ED visits declined. These results demonstrate that wastewater surveillance has the potential to complement conventional methods of influenza and RSV surveillance, detecting viral signals earlier and for a longer duration than do clinical data. Continued use of wastewater surveillance as a supplement to established surveillance systems such as ED visits might improve local understanding and response to seasonal respiratory virus outbreaks.

## Introduction

Wastewater surveillance has demonstrated benefit as a robust, highly adaptable platform for community-level surveillance for SARS-CoV-2 transmission (*1*). In considering expansion of wastewater surveillance to track other respiratory pathogens, influenza viruses and respiratory syncytial virus (RSV) are two potential candidates: both viruses are quantifiable in wastewater, associated with substantial morbidity and mortality each year, and spread during annual winter outbreaks that can vary in timing and duration (*2*,*3*). Wastewater surveillance, which is independent of health care access or testing biases, might help supplement outbreak data collected by traditional, clinical surveillance systems; data on usefulness of wastewater surveillance to track influenza and RSV, however, are limited. During the 2022–23 respiratory diseases season, the Wisconsin Department of Health Services tracked influenza A virus and RSV in Wisconsin’s three largest cities using wastewater surveillance data. Wastewater surveillance data for influenza A virus and RSV were compared with influenza- and RSV-associated emergency department (ED) visits, both descriptively and with basic correlation statistics, to broadly ascertain whether wastewater surveillance might be a useful, complementary surveillance tool for ongoing and future use in Wisconsin.

## Methods

### Data Sources

During August 2022–March 2023, wastewater samples were collected at least once weekly from approximately 40 wastewater treatment plants (treatment plants) as part of Wisconsin’s established wastewater surveillance system. Refrigerated samples were shipped overnight to either the Wisconsin State Laboratory of Hygiene or a University of Wisconsin-Milwaukee laboratory for processing; laboratories used different concentration and extraction methods, but all samples from a given treatment plant were processed by the same laboratory.* Established assays were used with CDC primers and probes^†^ to quantify concentrations (in gene copies per liter [gc/L]) of influenza A virus and RSV in samples (*4*). Weekly geometric mean concentrations were calculated in instances when more than one wastewater sample was tested from the same city during the same surveillance week. Concentration values were converted to the log(10) scale; a value of 1 gc/L was added to all values to allow for log(10) transformation of zero values.

ED visits for influenza and RSV were reported to the Electronic Surveillance System for Early Notification of Community-Based Epidemics (ESSENCE), the primary surveillance system used by CDC’s National Syndromic Surveillance Program. Among 139 EDs across Wisconsin, 129 (93%) report data to ESSENCE. ED visits with influenza or RSV diagnoses were identified using standard CDC definitions, which were based on the diagnosis code and clinical terms for influenza or RSV, respectively, in discharge notes.^§^ ED visit counts were summed for each surveillance week.

A valid comparison of the wastewater and ED visit surveillance systems required that the populations covered be geographically similar. Wastewater surveillance data reflect contributions from persons within a sewershed (the geographic area contributing wastewater to a sampling location). ED visit data included the residential zip codes of patients. To identify overlapping population groups, sewershed boundaries were examined, and four treatment plants with sewershed boundaries were identified by visual inspection that were best aligned with zip code boundaries. These four treatment plants serviced the three most populous cities in Wisconsin (one in Green Bay, one in Madison, and two in Milwaukee) and provided an average of 1.7–3.2 samples per week. Thus, only wastewater surveillance data from the four treatment plants servicing these three cities were included; ED visit data were only included in the analysis if they were 1) linked to patients with a Green Bay, Madison, or Milwaukee residential zip code, and 2) reported by a Wisconsin ED during August 2022–March 2023.

### Statistical Analyses

Data on wastewater surveillance viral concentrations and ED visits were paired by surveillance week, and Kendall’s tau (a nonparametric measure of correlation) was used to assess statistical agreement between the surveillance systems. Kendall’s tau, which has been applied in previous wastewater surveillance literature, was used to quantify the relationship between the two data sources; higher values indicate a stronger correlation. R software (version 4.1.3; R Foundation) was used to conduct all statistical analyses. This activity was reviewed by CDC and conducted consistent with applicable federal law and CDC policy.^¶^

## Results

**FIGURE 1:**
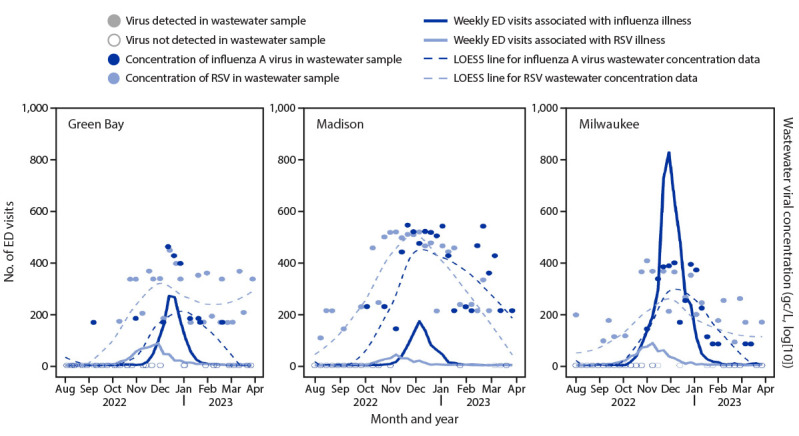
Respiratory syncytial virus–associated and influenza-associated emergency department visits* and wastewater concentrations^†^ for respiratory syncytial virus and influenza A virus — three Wisconsin cities, August 2022–March 2023^§^ **Abbreviations**: ED = emergency department; LOESS = locally estimated scatterplot smoothing; RSV = respiratory syncytial virus. * Collected from the Electronic Surveillance System for the Early Notification of Community-Based Epidemics (ESSENCE) and based on CDC-provided definitions. ^†^ Wastewater concentration values were log(10) and denoted in gene copies per L (gc/L); a value of 1 gc/L was added to all wastewater concentrations to allow for log(10) transformation of previously zero values. ^§^ LOESS lines are overlaid to display general trend in wastewater concentration data.

**FIGURE 2:**
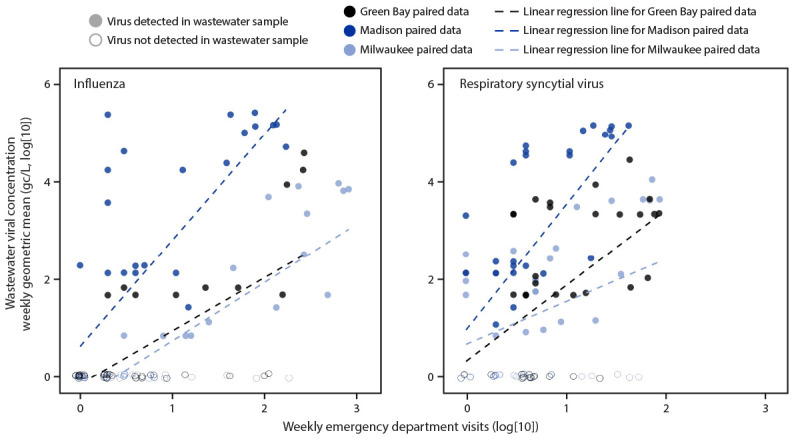
Correlation* between weekly emergency department visits and wastewater surveillance for influenza^†^ and respiratory syncytial virus^§^ — three Wisconsin cities, August 2022–March 2023 * For visual ease of comparison, data from both surveillance systems were transformed to the log(10) scale, and a value of 1 was added to all datapoints in both datasets (e.g., 1 case or 1 gc/L) to allow for log(10) transformation of previously zero values. Values along the 0 gc/L, log(10) line are jittered slightly to display the density of points. Linear regression lines are overlaid to display general trend in relationships between paired data. ^†^ Kendall’s tau values = 0.50 (Green Bay), 0.52 (Madison), and 0.63 (Milwaukee). ^§^ Kendall’s tau values = 0.37 (Green Bay), 0.49 (Madison), and 0.30 (Milwaukee).

A total of 6,271 influenza-associated ED visits and 1,518 RSV-associated ED visits were reported during August 2022–March 2023 (Table). According to both wastewater surveillance and ED data in each of the three cities, RSV peaked in early November, and influenza peaked approximately 1 month later (Figure 1). Wastewater samples from the three cities often tested positive for viral material in advance of rises in ED visit counts. Both influenza A virus and RSV detection persisted in sewersheds for up to 3 months after the associated ED visit trajectories had subsided. For both viruses, positive correlations were observed between paired wastewater surveillance and ED data in all three cities (Figure 2). Correlation values were higher for influenza than for RSV in all three cities. For Green Bay, Madison, and Milwaukee, Kendall’s tau values for influenza were 0.50, 0.52, and 0.63, respectively, and 0.37, 0.49, and 0.30, respectively, for RSV.

**TABLE:** Emergency department–based and wastewater-based surveillance for influenza and respiratory syncytial virus — Green Bay, Madison, and Milwaukee, Wisconsin, August 2022–March 2023

Characteristic	City			
	Green Bay	Madison	Milwaukee	Total
**Emergency department influenza data**				
No. of visits with illness meeting syndromic case definition*	1,251	805	4,215	**6,271**
Median age, yrs (IQR)	20 (6–44)	21 (7–45)	28 (14–48)	**26 (10–46)**
**Emergency department RSV data**				
No. of visits meeting syndromic case definition*	641	269	608	**1,518**
Median age, yrs (IQR)	2 (0–6)	1 (0–4)	3 (1–34)	**2 (0–9)**
**Wastewater data**				
No. of wastewater treatment plants serving city	1	1	2	**4**
Estimated population served by all wastewater treatment plants	189,000	345,000	1,085,941	**1,619,941**
Total no. of wastewater samples collected	61	78	111	**250**
Average no. of wastewater samples collected per wk	1.7	2.2	3.2	**2.4**

**Abbreviation**: RSV = respiratory syncytial virus.

* Diagnostic codes and clinical terms were based on *International Classification of Diseases, Tenth Revision, Clinical Modification* (ICD-10-CM), *International Classification of Diseases, Ninth Revision, Clinical Modification* (ICD-9-CM), and Systematized Nomenclature of Medicine–Clinical Terms (SNOMED CT). https://www.cdc.gov/ncird/surveillance/respiratory-illnesses/index.html#companion-guide

## Discussion

This analysis, which compared the results of wastewater surveillance and surveillance of ED visits for tracking influenza and RSV in three major Wisconsin cities during August 2022–March 2023, found a positive correlation between wastewater surveillance and ED visit data, findings that are consistent with previous analyses (*2*,*5*–*7*). The additional information concerning community levels of influenza and RSV circulation provided by wastewater surveillance might be beneficial for public health preparedness and response; for example, during the early stage of an epidemic wave (when case-based surveillance data are limited), it could be helpful to know when viral concentrations in wastewater start to increase. In addition, wastewater data might serve as supplementary input to forecasting models (*3*).

These findings are consistent with data from other reports showing that influenza virus and RSV are detected by wastewater surveillance in advance of rising clinical cases. Recent studies in Australia (*2*) and Canada (*8*) found that increasing concentrations of influenza virus and RSV in wastewater were strongly associated with increases in clinical cases 12–17 days later. Simple visual examination of the data in this current analysis suggested that influenza virus and RSV were generally detected in wastewater before significant increases in numbers of ED visits. However, this work was unable to determine the consistency or reliability of this lead time across cities or pathogens; for example, weekly aggregated data were too sparse to accurately calculate any time-shifted correlation coefficients. Future work could prioritize data collection and quantity in late summer and early fall seasons in anticipation of respiratory illness outbreaks to better ascertain these lead time values. Any advance warning provided by wastewater surveillance might provide health care and public health systems time to scale up capacity, ensure availability of treatment (e.g., antivirals), and promote preventive measures in advance of a clinical surge.

The observed persistence of influenza A virus and RSV detections in wastewater surveillance after the prevalence of ED visits declined likely reflects asymptomatic and mild illness outside the outbreak’s peak, as well as possible prolonged viral shedding; influenza A virus genetic material has been found in stool samples of infected persons for up to 3 weeks after infection (*9*). A better understanding of this persistence might reassure public health authorities that continued detection of viral material in wastewater (beyond an observed decline in clinical cases) is not necessarily indicative of a resurgent wave of disease, but rather an inherent characteristic of this type of surveillance.

### Limitations

The findings in this report are subject to at least four limitations. First, wastewater surveillance does not reflect disease patterns among residents not served by municipal wastewater infrastructure and thus would not be representative of households using septic systems. Similarly, wastewater surveillance would likely fail to collect data from diapered children, that might be important in surveillance for RSV, which predominates among young children. Second, surveillance data in both systems were based on weekly numbers, which might obscure nuanced outbreak or cluster patterns in a community. Third, the seasonal outbreak dynamics of influenza and RSV vary from year to year, and because this analysis only includes a small data set from one respiratory season, results presented here might not reflect disease patterns in all years. Finally, variability in wastewater concentrations for each community, and thus differences in temporal patterns between wastewater and ED data, could be attributable to a number of factors independent of the incidence in each community (e.g., wastewater temperature and pH and the presence of external chemical inhibitors) (*10*).

### Implications for Public Health

The positive correlation between wastewater surveillance and ED visit data for both influenza and RSV, along with the detection of the two pathogens in wastewater before increases in associated ED visits, suggests that wastewater surveillance might help supplement established clinical surveillance for these viruses. Public health practitioners should be aware of the long persistence of viral detection in wastewater surveillance. Incorporation of, and continued research into, the capabilities of wastewater surveillance might improve local public health agencies’ understanding of and response to seasonal respiratory virus disease outbreaks.

## Footnotes

*
https://doi.org/10.6084/m9.figshare.23669271

†
https://www.cdc.gov/coronavirus/2019-ncov/downloads/lab/multiplex-primers-probes-printer.pdf

§ Diagnostic codes and clinical terms were based on *International Classification of Diseases, Tenth Revision, Clinical Modification* (ICD-10-CM), *International Classification of Diseases, Ninth Revision, Clinical Modification* (ICD-9-CM), and Systematized Nomenclature of Medicine–Clinical Terms (SNOMED CT). https://www.cdc.gov/ncird/surveillance/respiratory-illnesses/index.html#companion-guide

¶ 45 C.F.R. part 46, 21 C.F.R. part 56; 42 U.S.C. Sect. 241(d); 5 U.S.C. Sect. 552a; 44 U.S.C. Sect. 3501 et seq.
